# Berberine Protects against High-Energy and Low-Protein Diet-Induced Hepatic Steatosis: Modulation of Gut Microbiota and Bile Acid Metabolism in Laying Hens

**DOI:** 10.3390/ijms242417304

**Published:** 2023-12-09

**Authors:** Chang Wang, Yitian Yang, Jinyan Chen, Xueyan Dai, Chenghong Xing, Caiying Zhang, Huabin Cao, Xiaoquan Guo, Guoliang Hu, Yu Zhuang

**Affiliations:** Jiangxi Provincial Key Laboratory for Animal Health, Institute of Animal Population Health, College of Animal Science and Technology, Jiangxi Agricultural University, No. 1101 Zhimin Avenue, Economic and Technological Development District, Nanchang 330045, China; 19179147923@163.com (C.W.); yangyitian0810@163.com (Y.Y.); jinyanchen2020@163.com (J.C.); 18722280937@163.com (X.D.); xch20175867@jxau.edu.cn (C.X.); zhangcaiying0916@163.com (C.Z.); chbin20020804@jxau.edu.cn (H.C.); xqguo20720@jxau.edu.cn (X.G.)

**Keywords:** berberine, high-energy and low-protein diet, fatty liver hemorrhagic syndrome, gut microbiota, bile acids

## Abstract

Berberine (BBR) is a natural alkaloid with multiple biotical effects that has potential as a treatment for fatty liver hemorrhagic syndrome (FLHS). However, the mechanism underlying the protective effect of BBR against FLHS remains unclear. The present study aimed to investigate the effect of BBR on FLHS induced by a high-energy, low-protein (HELP) diet and explore the involvement of the gut microbiota and bile acid metabolism in the protective effects. A total of 90 healthy 140-day-old Hy-line laying hens were randomly divided into three groups, including a control group (fed a basic diet), a HELP group (fed a HELP diet), and a HELP+BBR group (high-energy, high-protein diet supplemented with BBR instead of maize). Our results show that BBR supplementation alleviated liver injury and hepatic steatosis in laying hens. Moreover, BBR supplementation could significantly regulate the gut’s microbial composition, increasing the abundance of Actinobacteria and Romboutsia. In addition, the BBR supplement altered the profile of bile acid. Furthermore, the gut microbiota participates in bile acid metabolism, especially taurochenodeoxycholic acid and α-muricholic acid. BBR supplementation could regulate the expression of genes and proteins related to glucose metabolism, lipid synthesis (FAS, SREBP-1c), and bile acid synthesis (FXR, CYP27a1). Collectively, our findings demonstrate that BBR might be a potential feed additive for preventing FLHS by regulating the gut microbiota and bile acid metabolism.

## 1. Introduction

Fatty liver hemorrhagic syndrome (FLHS) is a metabolic disease characterized by a lipid metabolism disorder and accompanied by excessively high feed intake, increased body weight, individual obesity, a decreased egg production rate, and engorged, pale combs. Sudden death also occurs, and at necropsy, the syndrome is characterized by an excessive accumulation of fat within the abdominal cavity, a pale and fragile liver sometimes void of structural integrity, multifocal hemorrhages, and large blood clots in the abdominal cavity as a result of liver rupture [1,2,3]. It is typically brought on by the interaction of genetic and environmental variables, with an excessive intake of foods high in energy and low in protein (HELP) in the diet being a major contributing factor [4,5]. Recently, studies showed that a balanced diet structure and dietary additives can alleviate flora disorders and reduce the development of liver disease [6,7]. High-fat diets alter the gut microbiota ecosystem, resulting in severe liver diseases [8]. Nonalcoholic fatty liver disease (NAFLD), which has been renamed metabolic-associated fatty liver disease (MAFLD) in recent years [9], is a common chronic disease characterized by fatty deposits in hepatic cells and liver steatosis [10]. Liver steatosis is mainly characterized by an imbalance between fat secretion and metabolism after the transportation of fat to the liver [11]. NAFLD is associated with obesity, metabolic syndrome, and certain genetic variations, mainly due to the excessive accumulation of fat in the liver. When compared to healthy people, the gut microbiota of people with NAFLD shows significant structural and compositional abnormalities [12]. Reducing the ratio of Firmicutes to Bacteroidetes (F/B) is a crucial factor in improving NAFLD [13]. Therefore, maintaining a healthy gut microbiome may provide a fundamental strategy for preventing and treating FLHS, leading to a better understanding of the dietary interventions necessary to maintain gut health and minimize liver disease risks.

Gut microbiota plays a critical role in regulating fatty liver illnesses by producing bacterial metabolites, like short-chain fatty acids, secondary bile acids, and trimethylamine [14,15]. Bile acids (BAs), which the liver produces from cholesterol and secretes into the intestine, are metabolites of intestinal micro-organisms and can be effectively utilized by intestinal micro-organisms [16]. Furthermore, they can regulate glucose and lipid metabolism in the liver [17]. An increasing amount of experimental and clinical evidence indicates that BAs hold exceptional potential as a therapeutic approach for fatty liver disease, hypercholesterolemia, and metabolic diseases [18,19,20]. BA receptors are drawing attention as potential therapeutic targets for liver illnesses because BAs perform their numerous biological actions by attaching to their receptors [21]. More research has revealed that modifications to the gut microbiota affect the host’s BA profiles, most notably in the way taurine-conjugated BAs interact with the intestinal farnesol receptor (FXR) [22]. By lowering hepatic and plasma lipid levels, reducing inflammation, and enhancing insulin sensitivity, activation of the FXR may be able to reduce the symptoms of NAFLD [23]. Therefore, BAs can be used as a targeted therapeutic agent for fatty liver diseases. 

In recent years, there have been many studies on some natural products as ideal candidates for the treatment of NAFLD, and some of them are about to be approved. Berberine (BBR) is one of them, which has been clearly demonstrated to have significant therapeutic effects on NAFLD in some animal models and clinical studies [24]. However, whether BBR has a positive therapeutic effect on FLHS induced by HELP diets and its mechanism of action is yet to be clarified. BBR is a physiologically active isoquinoline alkaloid extracted mainly from the roots or stems of the herb Coptis chinensis. It possesses various biological effects, including immunomodulatory, antioxidant, and anti-inflammatory effects [25,26]. Recently, studies have revealed that BBR could directly regulate the composition of gut microbiota [27]. The study of Zhu et al. showed that 250 mg/kg BBR added to the feed of 1-day-old yellow-feathered broilers could reduce the Chao 1 and Shannon index, representing the microbial α-diversity and the abundance of the phylum Firmicutes. The genera Lachnospiraceae, Lachnoclostridium, Clostridiales, and Intestinimonas decreased, whereas the abundances of the phylum Bacteroidetes and the genus Bacteroides increased with BBR treatment. These results indicate that BBR improves the growth performance of broilers and reshapes their intestinal flora structure, playing a beneficial role [28]. However, although high doses of berberine can reduce intestinal inflammation in chickens and play a positive role, it can also increase the relative abundance of the family Enterobacteriaceae and decrease the relative abundance of the family Peptostreptococcaceae as well as the protective genera of the Ruminococcaceae and the Lachnospiraceae families, leading to a certain degree of dysbiosis [29]. BBR has also been demonstrated to be connected to the gut microbiota as a lipid-lowering medication by controlling BA turnover and activating subsequent ileal FXR signaling pathways [30]. BBR can reduce body weight, liver fat deposition, and triglyceride content in high-fat diet-induced obesity [31]. Li et al.’s studies have shown that BBR can ameliorate the progression of NAFLD by modulating the gut microbiota and improving the intestinal mucosal barrier function [32]. The protective effects of BBR on the gut microbiota and BA metabolism in FLHS, however, are the subject of few investigations. In the current study, we look at whether BBR can reduce FLHS symptoms caused by HELP in Hy-line brown laying hens. Then, through analyzing the gut microbiota and BA profile, we found that BBR could regulate glucose and lipid metabolism in laying hens by modifying the BA metabolism and gut flora. This research may offer fresh insight into how BBR improves FLHS by controlling the gut flora and BA metabolism.

## 2. Results

### 2.1. BBR Alleviated HELP-Induced Blood Lipid Metabolism and Hepatic Lipid Deposition

**Figure 1:**
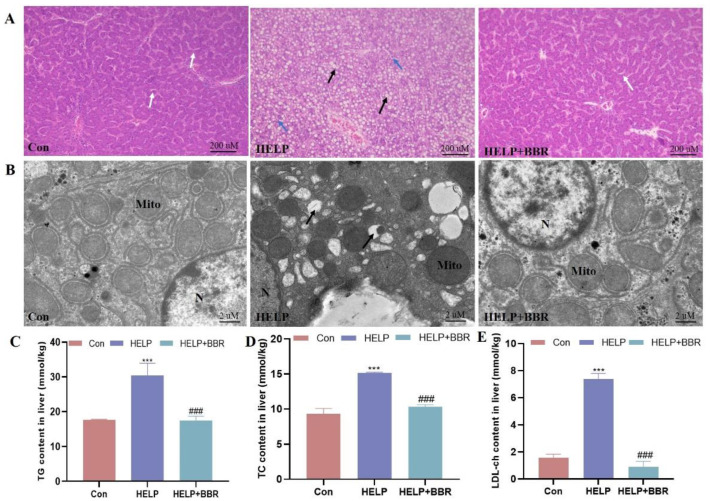
BBR alleviated HELP-induced blood lipid metabolism and hepatic lipid deposition. (**A**) The histological features (magnification: 200×, scale bar = 200 μm) and the frame indicate the location of characteristic lesions. White arrows represent interstitial hepatic cords, black arrows represent cellular steatosis, and blue arrows represent inflammatory cells; (**B**) Ultrastructural features (magnification: 1200×, scale bar = 2 μm), N: nucleus; Mito: mitochondria; (**C**) TG content; (**D**) TC content; (**E**) LDL-ch content. Data were represented as the mean ± SD. *** *p* < 0.001 vs. the Con group; ^###^ *p* < 0.001 vs. the HELP group. Con, control; HELP, high-energy low-protein diet; BBR, berberine. Below is the same.

As shown in Figure 1A, histological observation shows that there is a great deal of lipid-filled vacuoles in the liver cells of the HELP group, and the structure of the hepatic cord and hepatic lobule disappear, while the control group presents normal liver cell structure. The results obtained from transmission electron microscopy indicate that there is an increase in the number of fat vacuoles, and a decrease can be observed in the number of mitochondria within the HELP group (Figure 1B). Furthermore, the biochemical findings indicate that there is a noteworthy elevation in the levels of TG, TC, and LDL-ch (*p* < 0.001) within the HELP group. However, BBR intervention can mitigate pathological changes in liver tissue, alleviate fatty degeneration, and significantly decrease blood lipid levels (Figure 1C–E). 

### 2.2. BBR Alters the Composition of Gut Microbiota in HELP-Fed Laying Hens

**Figure 2:**
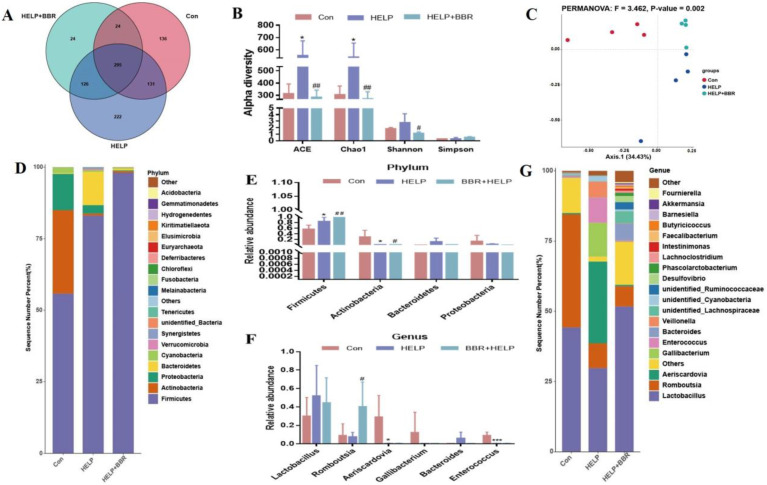
BBR alleviated HELP-induced intestinal microbiota dysbiosis. (**A**) Venn diagram showing the unique and shared OTUs in the diverse groups; (**B**) Alpha diversity; (**C**) Multiple-sample PCoA analysis; (**D**) Relative abundance of gut microbiota at the phylum level; (**E**) Relative abundance of the significantly altered bacteria at the phylum levels from the three groups; (**F**) Relative abundance of the significantly altered bacteria at the genus levels from the three groups; (**G**) Relative abundance of gut microbiota at the genus level. Data are represented as the mean ± SD. * *p* < 0.05 and *** *p* < 0.001 vs. the Con group; ^#^ *p*< 0.05 and ^##^ *p* < 0.01 vs. the HELP group.

Intestinal flora is a micro-ecosystem in the body, and its diversity is a key indicator of individual health. Therefore, the OTU species of each group were analyzed. There were 295 OTUs in the three groups, among which 136, 222, and 24 OTU species were unique in the Con group, the HELP group, and the BBR + HELP group, respectively (Figure 2A). The alpha diversity index is used to evaluate the richness and uniformity of micro-organisms in samples. As shown in Figure 2B, at the same sequencing depth, ACE and Chao1 were significantly increased in the HELP group and reduced in the HELP+BBR group. To measure the extent of similarity between the microbial communities, beta diversity was calculated using a weighted normalized UniFrac, and PCoA was performed (Figure 2C). The distance between the three groups was obviously separated, and PERMANOVA similarity analysis revealed that the three groups’ microbial distributions differed significantly from one another (F = 3.462, *p* = 0.002).

The flora in each group was examined at the phylum and genus levels to further assess the impact of BBR on intestinal flora. The results at the phylum level are shown in Figure 2D,E. The first four bacterial groups (Firmicutes, Actinobacteria, Bacteroidetes, and Proteobacteria) in the intestine of each group account for more than 90% of total phylum levels. Firmicute abundance was significantly upregulated, Actinobacterium and Proteobacterium abundances were significantly downregulated, and Bacteroidota abundance was raised in the HELP group. Compared to the HELP group, the addition of BBR in the HELP diet can further improve Firmicute abundance and reduce the abundance of Actinobacteria, Proteobacteria and Bacteroidetes. At the genus level, 21 species of bacteria, such as *Lactobacillus* and *Romboutsia,* were detected, among which *Lactobacillus* was the dominant bacteria with a relatively high abundance (Figure 2G,F). After BBR intervention, the abundance of *Lactobacillus* in the HELP group showed an increase by approximately 25% compared to the HELP group. Conversely, the abundance of *Lactobacillus* experienced a reduction of about 10% post-BBR intervention. *Bacteroides* were elevated in the HELP group, and bacterial abundance tended to be normal after BBR intervention. In addition, the abundance of *Romboutsia* flora showed a downward trend in the HELP group, but after the addition of BBR intervention, the abundance of flora increased significantly. The abundance of *Aeriscardovia*, *Gallibacterium*, and *Enterococcus* bacteria significantly decreased in the HELP group but did not improve after BBR intervention.

### 2.3. Regulation of BBR on Bile Acid Metabolism Disorder in Chicken Feces

**Figure 3:**
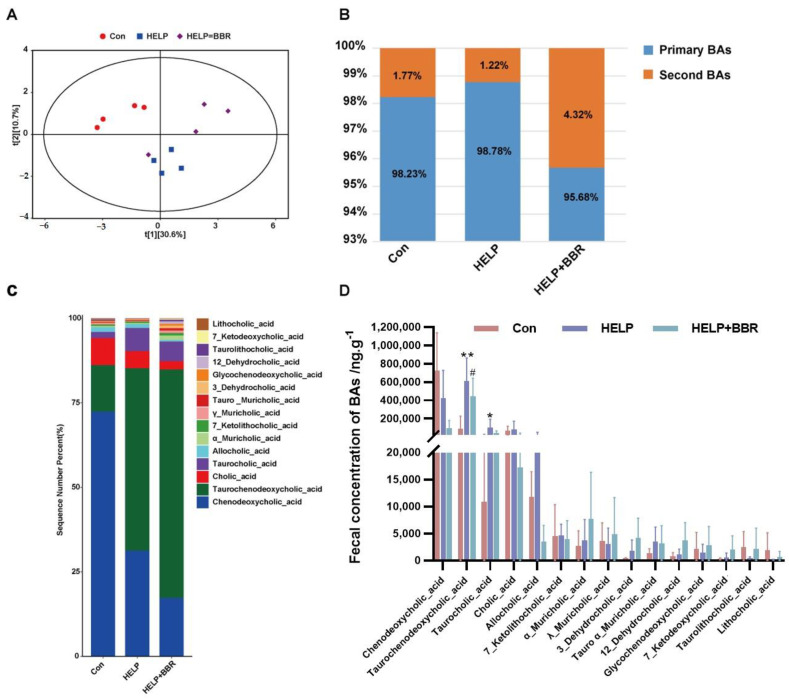
Regulation of BBR on bile acid metabolism disorder in chicken feces. (**A**) PLS-DA score plots for discriminating the fecal BA profiles from three groups; (**B**) Ratio of primary bile acid to secondary bile acid; (**C**) Composition of bile acid pool in feces; (**D**) Relative abundance of the significantly changed BAs from different groups. Data are represented as the mean ± SD. * *p* < 0.05 and ** *p* < 0.01 vs. the Con group; # *p* < 0.05 vs. the HELP group.

In the case of FLHS, the change in intestinal flora structure can lead to the abnormal metabolism of bile acid, and the intestinal immune balance and the stability of the intestinal barrier can be destroyed. BBR can further affect the metabolism of bile acid molecules by modifying the makeup of the intestinal flora. In Figure 3A, the control group and the HELP group can be observed to have different bile acid distributions. Specifically, the bile acid reaches the maximum distance in both groups but is congregated in two distinct quadrants for each group. However, when BBR is added to the HELP diet, the bile acid metabolism in the feces of FLHS laying hens changes. In addition, 15 bile acids in the intestinal contents of FLHS laying hens were accurately quantified and analyzed by the partial least-squares method. Ten free bile acids, five conjugated bile acids, eight primary bile acids, and five secondary bile acids were detected. Compared to the control group, the content of chenodeoxycholic acid declined, and the content of Taurochenodeoxycholic_acid, Taurocholic_acid, Cholic_acid, and Allocholic_acid increased in the HELP group (Figure 3B–D). However, bile acid content continued to decrease with the addition of BBR, indicating that BBR plays an important regulatory role in the bile acid metabolism of FLHS laying hens induced by HELP.

**Figure 4:**
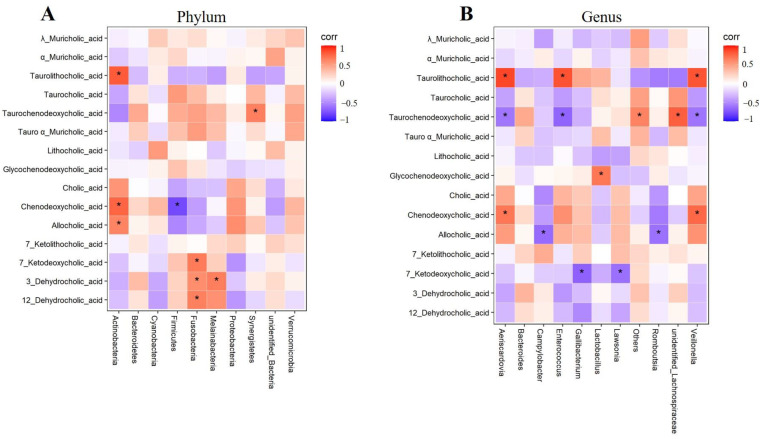
Heatmap analysis of the Spearman correlation of fecal BAs and gut microbiota. (**A**) Spearman correlation of fecal BA and gut microbiota at the phylum level; (**B**) Spearman correlation of fecal BA and gut microbiota at the genus level. Squares in red with an asterisk refer to a significant positive correlation, and squares in blue with an asterisk indicate a significant negative correlation. Data are represented as the mean ± SD. * *p* < 0.05 vs. the Con group.

Finally, this study analyzed the correlation between bile acid molecules and intestinal microflora at the phylum and genus levels, respectively. The results showed that bile acid was closely related to intestinal flora. At the phylum level (Figure 4A), the bacterial abundance of ileal *Actinobacteria* was significantly positively correlated with Chenodeoxycholic_acid, Taurolithocholic_acid, and Allocholic_acid. In addition, the content of *Fusobacteria* was positively correlated with 7_ketodeoxycholic_acid, 3_dehydrocholic_acid, and 12_dehydrocholic_acid. *Melainabacteria* and *Synergistetes* were positively correlated with 3_dehydrocholic_acid and taurochenodeoxycholic_acid, respectively. *Firmicuteria* was negatively correlated with Chenodeoxycholic_acid. At the genus level (Figure 4B), the bacterial abundance of *Aeriscardovia*, *Enterococcus,* and *Veillonella* were positively correlated with taurolithocholic_acid, and significantly negatively correlated with taurochenodeoxycholic_acid. *Campylobacter* and *Romboutsia* were negatively correlated with allocholic_acid, and *gallibacterium* and *lawsonia* were significantly negatively correlated with 7_ketodeoxycholic_acid.

### 2.4. BBR Alleviated HELP-Induced Abnormal Bile Acid Biosynthesis 

**Figure 5:**
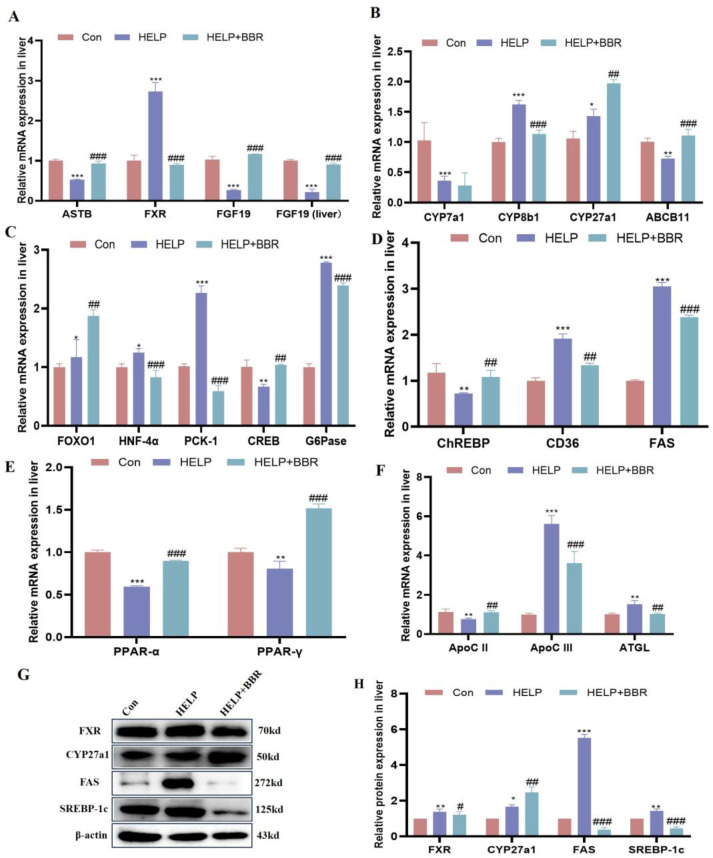
BBR alleviated HELP-induced lipid metabolism. (**A**) Bile acid biosynthesis-related mRNA expression level; (**B**) FXR receptor-related mRNA expression level; (**C**) Gluconeogenesis-related mRNA expression level; (**D**) lipid synthesis-related mRNA expression level; (**E**) lipid oxidation-related mRNA expression level; (**F**) Triglyceride hydrolysis-related mRNA expression level; (**G**) protein bands; (**H**) The results of gray-value analysis of protein bands. Data are represented as the mean ± SD. * *p* < 0.05, ** *p* < 0.01 and *** *p* < 0.001 vs. the Con group; ^#^ *p*< 0.05, ^##^ *p* < 0.01 and ^###^ *p* < 0.001 vs. the HELP group.

As shown in Figure 5A,B,G,H, compared to the control group, FXR receptor-related genes *ASBT*, *FGF19* (ileum), and *FGF19* (liver) mRNA levels declined (*p* < 0.001) in the HELP group. The FXR gene and protein were significantly increased in the HELP group. (*p* < 0.001; *p* < 0.01); bile acid synthesis genes *CYP7a1* and *ABCB11* mRNA levels were downregulated in the HELP group compared to the control group (*p* < 0.001; *p* < 0.05). Among them, *CYP8b1* and *CYP27a1* mRNA levels were upregulated in the HELP group compared to the control group (*p* < 0.001; *p* < 0.05). However, added BBR can significantly increase the expression of *ASBT*, *FGF19* (ileum), *FGF19* (liver), *CYP27a1,* and *ABCB11* mRNA levels (*p* < 0.001). The results of CYP27a1 protein were consistent with the results of gene expression.

### 2.5. BBR Alleviated HELP-Induced Abnormal Glucose and Lipid Metabolism

As shown in Figure 5C–F, compared to the control group involved in glucose metabolism genes, *FOXO1*, *HNF-4α*, *PCK-1,* and *G6Pase* mRNA levels increased (*p* < 0.001), but *CREB* mRNA level declined (*p* < 0.01) in the HELP group. For the added BBR in the HELP diet, the mRNA levels of *FOXO1*, *HNF-4α*, *PCK-1,* and *G6Pase* significantly declined (*p* < 0.01 or *p* < 0.001). Meanwhile, compared to the control group, lipid metabolism genes *chREBP*, *PPARα,* and *PPAR-γ* mRNA levels were reduced (*p* < 0.05), but *CD36* and *FAS* mRNA levels were upregulated (*p* < 0.01; *p* < 0.001) in the HELP group. Added BBR can significantly upregulate *chREBP*, *PPARα,* and *PPAR-γ* mRNA levels (*p* < 0.01 or *p* < 0.001) in the BBR+HELP group. At the same time, the expression of lipid synthesis proteins FAS and SREBP-1c in the HELP group significantly increased (*p* < 0.01; *p* < 0.001), and the addition of BBR could reduce the expression of proteins (*p* < 0.001) (Figure 5G,H). Additionally, compared to the control group, ApoC II mRNA levels were reduced (*p* < 0.01), but *ApoC III* and *ATGL* mRNA levels were upregulated (*p* < 0.01 or *p* < 0.001) in the HELP group. For added BBR in the HELP diet, the mRNA levels of *ApoC III* and *ATGL* were significantly reduced (*p* < 0.01), and *ApoC II* mRNA level was upregulated (*p* < 0.05). 

## 3. Discussion 

FLHS is a prevalent nutritional metabolic disease that affects laying hens, causing liver injury and a sudden decline in egg production during the peak laying period. However, a complete understanding of the underlying mechanisms concerning the development and advancement of FLHS remains to be fully elucidated. Recently, mounting evidence has suggested that the participation of the gastrointestinal–hepatic axis plays a crucial role in the emergence and advancement of NAFLD [33,34]. The gut microbiota, a fundamental component of the gut–liver axis, has been extensively recognized for its pivotal role in rewiring the energy metabolism of the host. In the present study, we successfully induced liver fat deposition in laying hens through a HELP diet (3100 kcal/kg, 12% crude protein), thus corroborating our previous study [5]. Furthermore, BBR has a direct impact on the microbiota in the intestine and controls the metabolism of bile acid through the regulation of the FXR signaling pathway. As a result, it enhances liver lipid metabolism in laying hens. 

The gastrointestinal–hepatic axis plays a key role in the development of NAFLD [35]. The expression of fatty acid synthase (FAS) and sterol regulatory element binding protein-1c (SREBP-1c) in the liver has been demonstrated to be inhibited by gut microbiota-derived metabolites derived from tryptophan metabolism. These metabolites control lipid metabolism by activating the hepatic aromatic hydrocarbon receptor (AHR) [36]. Accumulating evidence indicates the potential therapeutic effects of fecal microbiota transplantation in mitigating high-fat diet-induced steatohepatitis in murine models [37]. This effect may be due to an increase in beneficial gut microbiota, which improves the integrity of intestinal tight junctions and reduces the levels of lipopolysaccharides (LPS) [38]. In the present study, the intestinal microbiota structure in laying hens promotes hepatic lipid deposition, as demonstrated by alterations in intestinal microbiota α-diversity and β-diversity. Additionally, the supplementation of BBR has the potential to modulate the intestinal microbiota. 

Recent research has indicated that BBR may directly improve the function of the intestinal barrier, reduce inflammation, control the bile acid signal pathway, and regulate the axis of bacteria–gut–brain [39]. In both normal-chow-diet (NCD) and high-fat-diet (HFD) conditions, BBR had a significant impact on the makeup of the gut microbiota [40]. In particular, within the order Clostridiales, families Streptococcaceae, Clostridiaceae, and Prevotellaceae, as well as genera *Streptococcus* and *Prevotella*, BBR has shown a particular capacity to diminish the relative abundance of bacteria involved in the formation of branched-chain amino acids (BCAAs) [41]. In our results, according to the PCoA score plots, all three groups were significantly separated compared with themselves, indicating changes in bacterial communities. At the phylum level, the HELP group showed an increase in Firmicutes and Bacteroidetes. Firmicutes, as opposed to Bacteroidetes, digest sugar more effectively and favor energy resorption, the process by which surplus energy is converted to fat and stored in liver tissue when it is not used [42]. At the genus level, we found that the *Romboutsia* significantly increases in the BBR group compared with the HELP group. *Romboutsia* was dramatically reduced in obese individuals [43]. Other studies have connected *Romboutsia* to glycerophospholipids, which have been connected to obesity-related fatty liver disease [44,45]. Therefore, we speculate that BBR may improve lipid metabolism in HELP laying hens by regulating changes in the abundance of *Romboutsia*.

The field of metabolomics has recently gained popularity in biomedical research, although identifying many metabolites in untargeted metabolomics remains challenging. However, in nonalcoholic fatty liver disease, BA metabolism emerges as a desirable therapeutic target [46]. Therefore, in the current investigation, BA-targeted metabolomics was used to identify the alterations to BA in FLHS following BBR administration. A total of 15 bile acid metabolites were identified, among which the HELP group’s primary BA-to-secondary-BA ratio was higher than that of the Con group. After BBR treatment, the ratio of primary BA to secondary BA changed, manifested in Tauro chenodeoxycholic acid (TDCA) and Taurocholic acid (TCA). TUDCA, a derivative of Ursodeoxycholic acid (UDCA), was used for the treatment of liver dysfunction and increased HFD-induced obesity in a rat model [47,48]. Simultaneously, Cholic acid was converted into taurocholic acid, which could affect the metabolism of lipids and lipoproteins, glycolysis and gluconeogenesis, and fatty acid production [49]. These findings align with our experimental results that suggest that when FLHS occurs in laying hens, the body can relieve liver damage by secreting UDCA and TCA. However, additional supplementation of BBR can exogenously reduce damage and reduce the secretion of UDCA and TCA. Furthermore, by regulating the intestinal transit of bile acids, the gut microbiota can maintain bile acid homeostasis and regulate bile acid metabolism. According to earlier studies, gut microbes deconjugate primary bile acids generated from the host using bile salt hydrolases found in Actinobacteria [50]. At the phylum level, our analysis also revealed a substantial positive correlation between Actinobacteria and Chenodeoxycholic acid, Taurolithocholic acid, and Allocholic acid. Likewise, Aeriscardoviay was inversely connected with Taurochenodeoxychlic acid at the genus level but had a positive correlation with Chenodeoxycholic acid and Taurolithocholic acid.

By activating FXR and TGR5, BAs function as signal molecules that regulate not only their biosynthesis but also vital metabolic processes [51,52]. Therefore, we investigated whether the FXR signaling pathway is involved in the effects on BA production of the enhanced intestinal microbiota caused by BBR. Our findings in the current study showed that BBR stimulated liver FXR signaling to affect hepatic BA problems, and downstream bile acid synthesis-related genes (*CYP27a1* and *ABCB11* (*p* < 0.01)) are markedly upregulated in the BBR group, indicating that BBR can promote bile acid metabolism. Recent investigations have highlighted the significance of FXR as a critical therapeutic target in NAFLD, with encouraging findings showcasing the substantial amelioration of pathological manifestations in patients with NASH upon treatment with FXR agonists [53]. *Penthorum chinense* Pursh extract promotes bile acid biosynthesis and further reduces NAFLD in mice that are fed a high-cholesterol diet by promoting the production of the enzymes *CYP7a1* and *CYP8b1* and activating the liver’s FXR receptor [54]. By contrast, in our results, we found that BBR can reduce the expression of *CYP7a1* and *CYP8b1* and inhibit the activation of *FXR*. Moreover, based on Chao Yang et al., 28 weeks of FLA treatment substantially decreases the development of NASH in NAFL-model mice fed an HFD. These advantageous effects can be attributed to the regulation and enhancement of gut flora- and microbiota-related BAs, which in turn activate the intestinal FXR-FGF15 and TGR5-NF-κB pathways [55]. Additionally, Triglyceride and fatty acid metabolism are regulated by FXR. Haczeyni et al. demonstrated that Obeticholic acid reduced SREBP-1c expression and increased PPARα expression through the hepatic FXR signaling pathway in the liver of HFD mice [56]. Moreover, PPARγ and SREBP-1c expression are suppressed, and PPARα expression is increased by FXR, which is one strategy for preventing fat accumulation [57]. In the present study, BBR downregulated the glucose metabolism genes (*FOXO1, HNF-4α, PCK-1*, and *G6Pase* (*p* < 0.001)) and downstream lipogenic genes (*FAS* and *CD36* (*p* < 0.001)), as well as upregulating the expression of *PPARα*, *PPARγ*, and downregulating downstream triglyceride hydrolysis genes (*ApoC III* and *ATGL* (*p* < 0.01)). Thus, from our results that detect the genes and proteins of glucose and lipid metabolism downstream of the FXR signaling pathway, it is also shown that BBR has a positive therapeutic effect on liver lipid deposition caused by HELP.

## 4. Materials and Methods

### 4.1. Animals and Experimental Design

This study was approved by the Committee for the Care and Use of Experimental Animals at Jiangxi Agricultural University (No: JXAULL-202218). All experimental procedures adhered to the standards established by Jiangxi Agricultural University’s Experimental Animal Care and Use Committee. A total of 90 Hy-line laying hens, aged 140 days and in good health, were randomly distributed among three groups: control (Con), high-energy low-protein (HELP), and berberine (BBR)+ HELP. The diets provided to the laying hens in these groups comprised the basal diet, HELP diet, and HELP diet enriched with 100 mg/kg BBR, respectively. The experiment lasted for 140 days, during which all groups of hens received sufficient food and water. The nutritional requirements for the hens’ baseline diet were formulated in accordance with the standard guidelines specified by the National Research Council (1998). The HELP diet differed only in its energy and protein standards. The composition of both the basal and HELP diets can be found in Table 1.

After the 140-day experiment, each of the hens underwent anesthesia induced by an intravenous injection of sodium pentobarbital, administered at a dosage of 50 mg/kg following a 12-hour fast. Subsequently, samples of liver, distal ileum tissue, and ileal feces were carefully preserved at temperatures of −20 °C or −80 °C. Additionally, a segment of the liver tissue was preserved in a solution containing 4% paraformaldehyde to facilitate the examination of the corresponding indicators.

### 4.2. Histopathological Examination

The specific experimental method is consistent with Gao et al. [58]. The liver tissue specimens were washed with normal saline and then fixed in a 4% paraformaldehyde solution. After one week, the samples were routinely embedded and sliced (5 μm) and stained with hematoxylin and eosin (H&E). Afterward, pathological sections were observed using an optical microscope, and photographs were taken.

### 4.3. Determination of Liver Biochemical Indexes

We assessed the levels of triglyceride (TG), total cholesterol (TC), and low-density lipoprotein cholesterol (LDL-ch). In short, an appropriate amount of liver tissue (tissue weight: homogenate medium = 1:9) was weighed to prepare tissue homogenate. The homogenate was then centrifuged at 2500 rpm for 10 min to obtain the supernatant for testing, following the instructions provided by the manufacturer, the Nanjing Jiancheng Bioengineering Institute (based in Nanjing, China).

### 4.4. Sequencing of 16S rDNA 

The total microbial genomic DNA from the hen’s ileum feces was extracted and sent to BIOTREE (Shanghai, China). The DNA concentration and purity were assessed. A total of 1 ng/μL DNA concentration was selected as the template to amplify the V3–V4 fragment and the 16S rDNA gene using forward primer (5-CCTACGGGNGGCWGCAG-3) and reverse primer (5-GACTACHVGGG TATCTAATCC-3), and specific primers and high-fidelity enzymes were selected for PCR amplification [59]. The PCR amplification of the 16S rDNA gene was performed as follows: initial denaturation at 95 °C for 3 min, followed by 27 cycles of denaturing at 95 °C for 30 s, annealing at 55 °C for 30 s, and extension at 72 °C for 45 s, and single extension at 72 °C for 10 min, and ending at 4 °C. The PCR mixtures contained 5 × Trans Start Fast Pfu buffer 4 μL, 2.5 mM dNTPs 2 μL, forward primer (5 μM) 0.8 μL, reverse primer (5 μM) 0.8 μL, Trans Start Fast Pfu DNA Polymerase 0.4 μL, template DNA 10 ng, and ddH_2_O up to 20 μL. PCR reactions were performed in triplicate. The PCR product was extracted from 2% agarose gel and purified using the AxyPrep DNA Gel Extraction Kit (Axygen Biosciences, Union City, CA, USA) according to the manufacturer’s instructions and quantified using Quantus™ Fluorometer (Promega, WI, USA). Purified amplicons were pooled in equimolar, and paired-end sequenced (2 × 300) on an Illumina MiSeq platform (Illumina, San Diego, CA, USA). The 16S rDNA gene sequencing reads were demultiplexed, quality-filtered by Trimmomatic, and merged by FLASH with the following criteria: (i) 300 bp reads were truncated at any site receiving an average quality score of <20 over a 50 bp sliding window, and truncated reads shorter than 50 bp were discarded. Reads containing ambiguous characters were also discarded; (ii) only overlapping sequences longer than 10 bp were assembled according to their overlapped sequence. The maximum mismatch ratio of the overlap region was 0.2. Reads that could not be assembled were discarded; (iii) samples were distinguished according to the barcode and primers, and the sequence direction was adjusted for exact barcode matching and 2 nucleotide mismatches in primer matching.

Operational taxonomic units (OTUs) with 97% similarity cutoff were clustered using UPARSE (version 7.1, http://drive5.com/uparse/, 6 August 2022), and chimeric sequences were identified and removed. The taxonomy of each OTU representative sequence was analyzed by the RDP Classifier (http://rdp.cme.msu.edu/, 6 August 2022) against the 16S rDNA database (e.g., Silva 132/16s_bacteria) using a confidence threshold of 0.7.

### 4.5. Quantitative Analysis of BA Profile in Ileal Feces 

The concentration of BA in the samples was determined using UPLC–TQMS, a method developed in a previous study [60,61]. In short, 1000 μL of formic acid extract containing 0.1% formic acid was added to 25 mg of fecal samples, vortexed, and mixed under ice-water bath conditions, ground ultrasonically, left to stand at −40 °C, and centrifuged. Finally, the supernatant was extracted for UPLC–TQMS analysis. 

### 4.6. Quantitative Real-Time PCR Analysis 

Total RNA was extracted from liver tissues using the Trizol reagent (Takara, Dalian, China), as directed by the manufacturer. The RNA was then dissolved in 40 μL of clean, diethyl pyrocarbonate-treated water and stored at −80 °C. A biophotometer (Eppendorf, Germany) and agarose gel electrophoresis were used to evaluate the quantity and quality of the RNA. A One-Step gDNA Removal and cDNA Synthesis SuperMix kit from TransGen, Beijing, China, was used for the reverse-transcription (RT) procedure. The Anchored Oligo (dT)18 Primer, 10 μL of 2XES Reaction Mix, 1 μL of RT Enzyme Mix, 1 μL of gDNA Remover, and 7 μL of RNase-free ddH_2_O and cDNA made up the 20 μL of RT reactions. The reaction was carried out at 42 °C for 15 min, followed by 85 °C for 5 s, and finally, 4 °C indefinitely. The resulting cDNA was stored at −20 °C for real-time PCR.

The acquired chicken gene sequences and NCBI GenBank accession numbers are shown in Table 2. Shanghai Bioengineering Co., Ltd. was tasked with creating the primers for the housekeeping gene actin using the Primer Express 3.0 program. The ABI Quant Studio 7 Flex PCR apparatus was used to carry out the experiment. The reaction process was carried out in the following steps: predenaturation at 95 °C for 30 s, denaturation at 95 °C for 5 s, and annealing at 60 °C for 34 s. A total of 40 cycles were carried out. The 2^−ΔΔct^ approach was used to determine the relative mRNA levels.

### 4.7. Western Blot Analysis

An appropriate amount of liver tissue was taken and homogenized by adding lysate tissue. The tissue homogenate was then centrifuged at 4 °C at 15,000 rpm for 10 min. The total protein content of the liver samples was determined using the BCA protein detection kit (Solarbio, Beijing, China). The primary antibodies of FXR (1:1000) (Cat No. TD12402S) and CYP27a1 (1:1000) (Cat No. T58677S) were procured from ABMART. SREBP-1c (Cat No. 14088-1-AP), FAS (1:1000) (Cat No. 60196-1-lg), and β-actin (1:5000) (Cat No. 81115-1-RR) were procured from Proteinates. The Bio-Rad ChemiDoc Touch imager (Bio-Rad ChemiDoc Touch, California, USA) captured the signal. Finally, the gray cost of the corresponding protein was analyzed using ImageJ software V1.8.0 (ImageJ, RRID:SCR_003070).

### 4.8. Statistical Analysis

The data were presented as mean ± standard deviation (SD). GraphPad Prism 9.0 (GraphPad Inc., La Jolla, CA, USA), Microsoft Excel 2019, and SPSS version 26.0 (SPSS Inc., Chicago, IL, USA) were utilized for data analysis. One-way analysis of variance (ANOVA) and post hoc testing, known as the least significant difference (LSD), was employed in the data analysis. Statistical significance was set at a *p*-value of less than 0.05 (*p* < 0.05). Correlation analysis between fecal BA-related bacteria was performed using Spearman’s rank correlation, with a coefficient of > |0.5|, as well as a *p*-value < 0.05.

## 5. Conclusions

In summary, our study suggests that BBR has a potential therapeutic effect on FLHS, potentially through modulating the gut microbiota and regulating lipid metabolism. This novel finding provides important insights into the pathogenesis of FLHS and may contribute to the development of new treatments for FLHS.

## Figures and Tables

**Table 1:** Composition and nutrient levels of diets (air-dry basis) %.

Composition of Diet %	Control Group	HELP Group
Corn	64.00	70.00
Wheat Bran	2.00	1.20
Soybean Meal	24.00	14.58
Fat-Soybean Oil	0	4.22
Calcium	8.00	8.00
* Premix	2.00	2.00
Total	100.00	100.00
**Nutrient level**		
Crude Protein CP	15.86	12.00
Available Phosphorus (AP)	0.51	0.46
Arginine	1.03	0.74
Methionine	0.37	0.32
Valine	0.77	0.58
Metabolic Energy (kcal/kg)	2678.99	3100.00
Met + Cys	0.67	0.56

* The ingredient of premix: The ingredient of premix: multiple vitamins, 30 mg; cupric sulfate, 4.6 mg; ferrous sulfate, 28.4 mg; manganous sulfate, 35.46 mg; zinc sulfate, 76 mg; zeolite powder, 6 mg; sodium selenite, 5 mg; anti-oxidizing quinolone, 50 mg; choline, 90 mg; bacitracin zinc, 26.7 mg; bran, 350 mg; methionine, 100 mg.

**Table 2:** Gene primer sequence and their GenBank accession number.

Gene Name	Accession Number	Primer Sequences (5′ to 3′)
*FXR*	AF49249.7	Forward: CTCTCGCAAAATGGGGCAGT
		Reverse: CGCGGGAATTCGATTGGC
*ASBT*	AB970773.1	Forward: ACCATGAAATTGAAACAAGAGTGAA
		Reverse: TGGGATAACTTTAGCCTGTCCA
*FGF19*	NM_204674.3	Forward: GCCAGAGGTCTACTCATCGC
		Reverse: ACCTGCAACATTCTGCGGTA
*CYP7a1*	NM_001001753.2	Forward: GCTCCGCATGTTCCTGAATG
		Reverse: ATGGTGTTAGCTTGCGAGGC
*CYP8b1*	NM_001389480.2	Forward: TACCAAGGGACAGGGAACAAGGAG
		Reverse: GGAGGCAACACGGCATAGGC
*ABCB11*	XM_046921923.1	Forward: ATCTTGGCCATCCAGCAAGG
		Reverse: ACTGGCTCTTGCTCAACAACACC
*G6Pase*	BM439740.1	Forward: TCCAGCACATCCACTCCATCTACC
		Reverse: TCAACACCAAGCATCCGCAGAAG
*CREB*	CAJNRD030001119.1	Forward: ACCTGCCATTGCCACTGTTACG
		Reverse: CTCCATCCGTGCCGTTGTTAGAC
*FOXO1*	NM_204328.2	Forward: ACACAGTGAACCCCATGTCA
		Reverse: AGGGGCATACGGGTTCATAG
*HNF-4α*	AY700581.1	Forward: AGGATGTCTTGCTGCTAGGG
		Reverse: GCAGGCGTATTCATTGTCGT
*FAS*	AB495724.1	Forward: ACTGTGGGCTCCAAATCTTCA
		Reverse: CAAGGAGCCATCGTGTAAAGC
*ATGL*	EU240627.2	Forward: GCTGATCCAGGCCTGTGTCT
		Reverse: TGGAGGTATCTGCCCACAGTAGA
*PPAR-γ*	AB045597.1	Forward: CACTGCAGGAACAGAACAAAGAA Reverse: TCCACAGAGCGAAACTGACATC
*CD36*	NM_001030731.1	Forward: CTGGGAAGGTTACTGCGATT Reverse: GCGAGGAACTGTGAAACGATA
*ChREBP*	EU152408.1	Forward: GATGAGCACCGCAAACCAGAGG Reverse: TCGGAGCCGCTTCTTGTAGTAGG
*PPAR-α*	AF163809.1	Forward: GACACCCTTTCACCAGCATC Reverse: CCCTTACAACCTTCACAAGCA
*ApoC II*	CM040951.1	Forward: CCTCCCAGCTCACCCAATT Reverse: CAGGATCCCGGTGTAAGTCA
*ApoC III*	NM_001302127.2	Forward: AAGGTGCAGGAGTACGTCAA Reverse: GCGTTGTCTGACAGCCATTT
*CYP27a1*	XM_040676620.2	Forward: CTTCCCCAAGAACACCCTCT Reverse: AAGGGATGGAGCTGAAAGGG
*PCK-1*	NM_205471.2	Forward: TCAACACCAGATTCCCAGGC Reverse: CCTCATGCTAGCCACCACAT
*β-actin*	L08165.1	Forward: ATTGCTGCGCTCGTTGTT Reverse: CTTTTGCTCTGGGCTTCA

## Data Availability

The datasets analyzed during the current study are available from the corresponding author upon reasonable request.

## References

[1] Chen W., Shi Y., Li G., Huang C., Zhuang Y., Shu B., Cao X., Li Z., Hu G., Liu P. (2021). Preparation of the peroxisome proliferator-activated receptor alpha polyclonal antibody: Its application in fatty liver hemorrhagic syndrome. Int. J. Biol. Macromol..

[2] Grimes J.L., Maurice D.V., Lightsey S.F., Bridges W.J. (1991). Research note: Relationship of comb color to liver appearance and fat content in Single Comb White Leghorn laying hens. Poult. Sci..

[3] Rozenboim I., Mahato J., Cohen N.A., Tirosh O. (2016). Low protein and high-energy diet: A possible natural cause of fatty liver hemorrhagic syndrome in caged White Leghorn laying hens. Poult. Sci..

[4] Diaz G.J., Squires E.J., Julian R.J. (1999). The use of selected plasma enzyme activities for the diagnosis of fatty liver-hemorrhagic syndrome in laying hens. Avian Dis..

[5] Zhuang Y., Xing C., Cao H., Zhang C., Luo J., Guo X., Hu G. (2019). Insulin resistance and metabonomics analysis of fatty liver haemorrhagic syndrome in laying hens induced by a high-energy low-protein diet. Sci. Rep..

[6] Ma S., Sun Y., Zheng X., Yang Y. (2021). Gastrodin attenuates perfluorooctanoic acid-induced liver injury by regulating gut microbiota composition in mice. Bioengineered.

[7] Han K.H., Ohashi S., Sasaki K., Nagata R., Pelpolage S., Fukuma N., Reed J.D., Shimada K.I., Kadoya N., Fukushima M. (2020). Dietary adzuki bean paste dose-dependently reduces visceral fat accumulation in rats fed a normal diet. Food Res. Int..

[8] Yu J., Marsh S., Hu J., Feng W., Wu C. (2016). The pathogenesis of nonalcoholic fatty liver disease: Interplay between diet, gut microbiota, and genetic background. Gastroenterol. Res. Pract..

[9] Eslam M., Sanyal A.J., George J. (2020). MAFLD: A consensus-driven proposed nomenclature for metabolic associated fatty liver disease. Gastroenterology.

[10] Marchesini G., Brizi M., Bianchi G., Tomassetti S., Bugianesi E., Lenzi M., McCullough A.J., Natale S., Forlani G., Melchionda N. (2001). Nonalcoholic fatty liver disease: A feature of the metabolic syndrome. Diabetes.

[11] Vuppalanchi R., Chalasani N. (2009). Nonalcoholic fatty liver disease and nonalcoholic steatohepatitis: Selected practical issues in their evaluation and management. Hepatology.

[12] Long X., Liu D., Gao Q., Ni J., Qian L., Ni Y., Fang Q., Jia W., Li H. (2021). Bifidobacterium adolescents alleviates liver steatosis and steatohepatitis by increasing fibroblast growth factor 21 sensitivity. Front. Endocrinol..

[13] Wang B., Jiang X., Cao M., Ge J., Bao Q., Tang L., Chen Y., Li L. (2016). Altered fecal microbiota correlates with liver biochemistry in nonobese patients with Non-alcoholic fatty liver disease. Sci. Rep..

[14] Abilos A., de Gottardi A., Rescigno M. (2020). The gut-liver axis in liver disease: Pathophysiological basis for therapy. J. Hepatol..

[15] Juarez-Hernandez E., Chavez-Tapia N.C., Uribe M., Barbero-Becerra V.J. (2016). Role of bioactive fatty acids in nonalcoholic fatty liver disease. Nutr. J..

[16] Presides G.A., Keaton M.A., Campeau P.M., Bessard B.C., Conner M.E., Hotez P.J. (2014). The undernourished neonatal mouse metabolome reveals evidence of liver and biliary dysfunction, inflammation, and oxidative stress. J. Nutr..

[17] Ichimura-Shimizu M., Watanabe S., Kashirajima Y., Nagatomo A., Wada H., Tsuneyama K., Omagari K. (2022). Dietary cholic acid exacerbates liver fibrosis in NASH model of sprague-dawley rats Fed a high-fat and high-cholesterol diet. Int. J. Mol. Sci..

[18] Huang W., Ma K., Zhang J., Qatan ani M., Cuvillier J., Liu J., Dong B., Huang X., Moore D.D. (2006). Nuclear receptor-dependent bile acid signaling is required for normal liver regeneration. Science.

[19] Safari H., Kaczorowski N., Felder M.L., Brannon E.R., Varghese M., Singer K., Eniola-Adefeso O. (2020). Biodegradable, bile salt microparticles for localized fat dissolution. Sci. Adv..

[20] Sinal C.J., Tohkin M., Miyata M., Ward J.M., Lambert G., Gonzalez F.J. (2000). Targeted disruption of the nuclear receptor FXR/BAR impairs bile acid and lipid homeostasis. Cell.

[21] Li R., Palmiotti A., de Vries H.D., Hovingh M.V., Koehorst M., Mulder N.L., Zhang Y., Kats K., Bloks V.W., Fu J. (2021). Low production of 12alpha-hydroxylated bile acids prevents hepatic steatosis in Cyp2c70(-/-) mice by reducing fat absorption. J. Lipid. Res..

[22] Park M.Y., Kim S.J., Ko E.K., Ahn S.H., Seo H., Sung M.K. (2016). Gut microbiota-associated bile acid deconjugation accelerates hepatic steatosis in ob/ob mice. J. Appl. Microbiol..

[23] Yao J., Zhou C.S., Ma X., Fu B.Q., Tao L.S., Chen M., Xu Y.P. (2014). FXR agonist GW4064 alleviates endotoxin-induced hepatic inflammation by repressing macrophage activation. World J. Gastroenterol..

[24] Tarantino G., Balsano C., Santini S.J., Brienza G., Clemente I., Cosimini B., Sinatti G. (2021). It is high time physicians thought of natural products for alleviating NAFLD. Is there sufficient evidence to use them?. Int. J. Mol. Sci..

[25] Pirillo A., Catapano A.L. (2015). Berberine, a plant alkaloid with lipid- and glucose-lowering properties: From in vitro evidence to clinical studies. Atherosclerosis.

[26] Song D., Hao J., Fan D. (2020). Biological properties and clinical applications of berberine. Front. Med..

[27] Habtemariam S. (2020). Berberine pharmacology and the gut microbiota: A hidden therapeutic link. Pharmacol. Res..

[28] Zhu C., Huang K., Bai Y., Feng X., Gong L., Wei C., Huang H., Zhang H. (2021). Dietary supplementation with berberine improves growth performance and modulates the composition and function of cecal microbiota in yellow-feathered broilers. Poult. Sci..

[29] Dehau T., Cherlet M., Croubels S., van Immerseel F., Goossens E. (2023). A high dose of dietary berberine improves gut wall morphology, despite an expansion of Enterobacteriaceae and a reduction in beneficial microbiota in broiler chickens. mSystems.

[30] Sun R., Yang N., Kong B., Cao B., Feng D., Yu X., Ge C., Huang J., Shen J., Wang P. (2017). Orally administered berberine modulates hepatic lipid metabolism by altering microbial bile acid metabolism and the intestinal FXR signaling pathway. Mol. Pharmacol..

[31] Noh J.W., Jun M.S., Yang H.K., Lee B.C. (2022). Cellular and molecular mechanisms and effects of berberine on obesity-induced inflammation. Biomedicines.

[32] Li D., Zheng J., Hu Y., Hou H., Hao S., Liu N., Wang Y. (2017). Amelioration of intestinal barrier dysfunction by berberine in the treatment of nonalcoholic fatty liver disease in rats. Pharmacogn. Mag..

[33] Zhu L., Baker S.S., Gill C., Liu W., Alkhouri R., Baker R.D., Gill S.R. (2013). Characterization of gut microbiomes in nonalcoholic steatohepatitis (NASH) patients: A connection between endogenous alcohol and NASH. Hepatology.

[34] Jiang D., Zhang J., Lin S., Wang Y., Chen Y., Fan J. (2021). Prolyl endopeptidase gene disruption improves gut dysbiosis and non-alcoholic fatty liver disease in mice induced by a high-fat diet. Front. Cell Dev. Biol..

[35] Aron-Wisnewsky J., Vigliotti C., Witjes J., Le P., Holleboom A.G., Verheij J., Nieuwdorp M., Clement K. (2020). Gut microbiota and human NAFLD: Disentangling microbial signatures from metabolic disorders. Nat. Rev. Gastroenterol. Hepatol..

[36] Krishnan S., Ding Y., Saedi N., Choi M., Sridharan G.V., Sherr D.H., Yarmush M.L., Alaniz R.C., Jayaraman A., Lee K. (2018). Gut microbiota-derived tryptophan metabolites modulate inflammatory response in hepatocytes and macrophages. Cell Rep..

[37] Zhou D., Pan Q., Shen F., Cao H.X., Ding W.J., Chen Y.W., Fan J.G. (2017). Total fecal microbiota transplantation alleviates high-fat diet-induced steatohepatitis in mice via beneficial regulation of gut microbiota. Sci. Rep..

[38] Li J., Li Y., Feng S., He K., Guo L., Chen W., Wang M., Zhong L., Wu C., Peng X. (2022). Differential effects of dietary white meat and red meat on NAFLD progression by modulating gut microbiota and metabolites in rats. Oxidative Med. Cell. Longev..

[39] Wang H., Zhang H., Gao Z., Zhang Q., Gu C. (2022). The mechanism of berberine alleviating metabolic disorder based on gut microbiome. Front. Cell. Infect. Microbiol..

[40] Yu M., Alimujiang M., Hu L., Liu F., Bao Y., Yin J. (2021). Berberine alleviates lipid metabolism disorders via inhibition of mitochondrial complex I in gut and liver. Int. J. Biol. Sci..

[41] Yue S.J., Liu J., Wang A.T., Meng X.T., Yang Z.R., Peng C., Guan H.S., Wang C.Y., Yan D. (2019). Berberine alleviates insulin resistance by reducing peripheral branched-chain amino acids. Am. J. Physiol.-Endocrinol. Metab..

[42] Wu G., Sun X., Cheng H., Xu S., Li D., Xie Z. (2022). Large yellow tea extract ameliorates metabolic syndrome by suppressing lipogenesis through SIRT6/SREBP1 pathway and modulating microbiota in leptin receptor knockout rats. Foods.

[43] Martinez-Cuesta M.C., Del C.R., Garriga-Garcia M., Pelaez C., Requena T. (2021). Taxonomic characterization and short-chain fatty acids production of the obese microbiota. Front. Cell. Infect. Microbiol..

[44] Zeng Q., Li D., He Y., Li Y., Yang Z., Zhao X., Liu Y., Wang Y., Sun J., Feng X. (2019). Discrepant gut microbiota markers for the classification of obesity-related metabolic abnormalities. Sci. Rep..

[45] Vazquez-Moreno M., Perez-Herrera A., Locia-Morales D., Dizzel S., Meyre D., Stearns J.C., Cruz M. (2021). Association of gut microbiome with fasting triglycerides, fasting insulin and obesity status in Mexican children. Pediatr. Obes..

[46] Zhao W.W., Xiao M., Wu X., Li X.W., Li X.X., Zhao T., Yu L., Chen X.Q. (2021). Ilexsaponin A (1) ameliorates diet-induced nonalcoholic fatty liver disease by regulating bile acid metabolism in mice. Front. Pharmacol..

[47] Jiang C., Xie C., Lv Y., Li J., Krausz K.W., Shi J., Brocker C.N., Desai D., Amin S.G., Bisson W.H. (2015). Intestine-selective farnesoid X receptor inhibition improves obesity-related metabolic dysfunction. Nat. Commun..

[48] Lin H., An Y., Tang H., Wang Y. (2019). Alterations of bile acids and gut microbiota in obesity induced by high fat diet in rat model. J. Agric. Food. Chem..

[49] Zhu K., Nie S., Gong D., Xie M. (2016). Effect of polysaccharide from Ganoderma atrum on the serum metabolites of type 2 diabetic rats. Food Hydrocoll..

[50] He Z., Ma Y., Yang S., Zhang S., Liu S., Xiao J., Wang Y., Wang W., Yang H., Li S. (2022). Gut microbiota-derived ursodeoxycholic acid from neonatal dairy calves improves intestinal homeostasis and colitis to attenuate extended-spectrum beta-lactamase-producing enteroaggregative Escherichia coli infection. Microbiome.

[51] Sirvent A., Claudel T., Martin G., Brozek J., Kosykh V., Darteil R., Hum D.W., Fruchart J.C., Staels B. (2004). The farnesoid X receptor induces very low-density lipoprotein receptor gene expression. FEBS Lett..

[52] Staels B., Fonseca V.A. (2009). Bile acids and metabolic regulation: Mechanisms and clinical responses to bile acid sequestration. Diabetes Care.

[53] Neuschwander-Tetri B.A., Loomba R., Sanyal A.J., Lavine J.E., Van Natta M.L., Abdelmalek M.F., Chalasani N., Dasarathy S., Diehl A.M., Hameed B. (2015). Farnesoid X nuclear receptor ligand obeticholic acid for non-cirrhotic, non-alcoholic steatohepatitis (FLINT): A multicentre, randomised, placebo-controlled trial. Lancet.

[54] Li X., Zhao W., Xiao M., Yu L., Chen Q., Hu X., Zhao Y., Xiong L., Chen X., Wang X. (2022). Penthorum chinense Pursh. extract attenuates non-alcholic fatty liver disease by regulating gut microbiota and bile acid metabolism in mice. J. Ethnopharmacol..

[55] Yang C., Wan M., Xu D., Pan D., Xia H., Yang L., Sun G. (2021). Flaxseed powder attenuates non-alcoholic steatohepatitis via modulation of gut microbiota and bile acid metabolism through gut-liver axis. Int. J. Mol. Sci..

[56] Haczeyni F., Poekes L., Wang H., Mridha A.R., Barn V., Geoffrey H.W., Ioannou G.N., Yeh M.M., Leclercq I.A., Teoh N.C. (2017). Obeticholic acid improves adipose morphometry and inflammation and reduces steatosis in dietary but not metabolic obesity in mice. Obesity.

[57] Hassan H.M., Guo H., Yousef B.A., Guerram M., Hamdi A.M., Zhang L., Jiang Z. (2016). Role of inflammatory and oxidative stress, cytochrome P450 2E1, and bile acid disturbance in rat liver injury induced by isoniazid and lipopolysaccharide cotreatment. Antimicrob. Agents. Chemother..

[58] Gao X., Liu P., Wu C., Wang T., Liu G., Cao H., Zhang C., Hu G., Guo X. (2019). Effects of fatty liver hemorrhagic syndrome on the AMP-activated protein kinase signaling pathway in laying hens. Poult. Sci..

[59] Huang F., Zheng X., Ma X., Jiang R., Zhou W., Zhou S., Zhang Y., Lei S., Wang S., Kuang J. (2019). Theabrownin from Pu-erh tea attenuates hypercholesterolemia via modulation of gut microbiota and bile acid metabolism. Nat. Commun..

[60] Zheng X., Huang F., Zhao A., Lei S., Zhang Y., Xie G., Chen T., Qu C., Rajani C., Dong B. (2017). Bile acid is a significant host factor shaping the gut microbiome of diet-induced obese mice. BMC Biol..

[61] Xie G., Wang Y., Wang X., Zhao A., Chen T., Ni Y., Wong L., Zhang H., Zhang J., Liu C. (2015). Profiling of serum bile acids in a healthy Chinese population using UPLC-MS/MS. J. Proteome Res..

